# Fruit shape diversity in the Brassicaceae is generated by varying patterns of anisotropy

**DOI:** 10.1242/dev.135327

**Published:** 2016-09-15

**Authors:** Tilly Eldridge, Łukasz Łangowski, Nicola Stacey, Friederike Jantzen, Laila Moubayidin, Adrien Sicard, Paul Southam, Richard Kennaway, Michael Lenhard, Enrico S. Coen, Lars Østergaard

**Affiliations:** 1 John Innes Centre, Norwich Research Park, Norwich NR4 7UH, UK; 2 Biosciences Eastern and Central Africa - International Livestock Research Institute (BecA-ILRI) Hub, P.O. Box 30709, Nairobi00100, Kenya; 3 Institute for Biochemistry and Biology, University of Potsdam, Potsdam 14476, Germany; 4 University of East Anglia, Norwich NR4 7TJ, UK

**Keywords:** Brassicaceae, *Capsella*, *Arabidopsis*, Fruit shape, Modelling, Anisotropic growth

## Abstract

Fruits exhibit a vast array of different 3D shapes, from simple spheres and cylinders to more complex curved forms; however, the mechanism by which growth is oriented and coordinated to generate this diversity of forms is unclear. Here, we compare the growth patterns and orientations for two very different fruit shapes in the Brassicaceae: the heart-shaped *Capsella rubella* silicle and the near-cylindrical *Arabidopsis thaliana* silique. We show, through a combination of clonal and morphological analyses, that the different shapes involve different patterns of anisotropic growth during three phases. These experimental data can be accounted for by a tissue-level model in which specified growth rates vary in space and time and are oriented by a proximodistal polarity field. The resulting tissue conflicts lead to deformation of the tissue as it grows. The model allows us to identify tissue-specific and temporally specific activities required to obtain the individual shapes. One such activity may be provided by the valve-identity gene *FRUITFULL*, which we show through comparative mutant analysis to modulate fruit shape during post-fertilisation growth of both species. Simple modulations of the model presented here can also broadly account for the variety of shapes in other Brassicaceae species, thus providing a simplified framework for fruit development and shape diversity.

## Abstract (precis)

**Summary:** The diversity of fruit shape in the Brassicaceae family is based on local variation of directional growth that alters during developmental phases.

## INTRODUCTION

Despite the great diversity in plant organ shapes, it has been proposed that common principles may underlie shape determination (reviewed by Sluis and Hake, 2015). Based on inheritance studies, it was recognised from the early days of genetics that genes regulate organ size and shape determination (Emerson and East, 1913; Freeman, 1919; Dale, 1925; Sinnott and Kaiser, 1934). In fact, Sinnott's work on *Cucurbita pepo* fruit growth showed that it is possible to genetically differentiate between activities that regulate shape and activities promoting growth (Sinnott, 1935). More recently, key genetic factors involved in determining fruit shape in domesticated fruit crops such as tomato, melon and pepper have been uncovered (Tanksley, 2004; Paran and van der Knaap, 2007; Monforte et al., 2014). Furthermore, tissue-level models of leaf and petal growth have led to the suggestion that shape depends on patterns of specified anisotropic growth that are oriented by a polarising field (Green et al., 2010; Kuchen et al., 2012; Sauret-Gueto et al., 2013). Conflicts generated by regions growing with different rates or orientations lead to changes in curvature and shape. However, it is unclear whether such models could account for the growth patterns and diversity of 3D fruit shapes. Here, we address this problem by analysing the growth and development of two very different fruit shapes in the Brassicaceae.

Although the overall composition and organisation of fruit tissues are highly conserved among members of the Brassicaceae family, huge diversity exists in their shape, which include, for example, cylindrical, disc-formed, spherical and heart-shaped structures (Langowski et al., 2016). In many cases it is not immediately evident what advantages the different shapes provide for fitness and dispersal. It is also unclear how such variation in form can evolve when coordination of tissue growth and specification is of such pivotal importance for timely development and seed release. Comparative analysis of fruit development in well-studied species with different fruit shapes, such as *Arabidopsis thaliana* and its relative *Capsella rubella*, might provide a framework for addressing these issues.

**Fig. 1.**
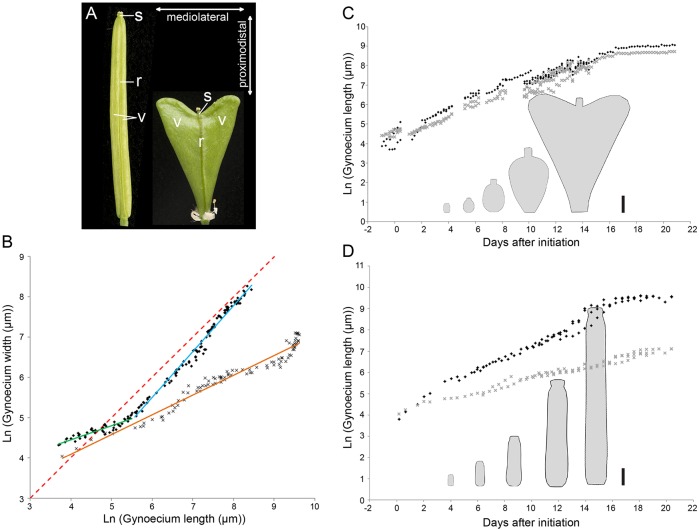
**Fruit growth analysis.** (A) Mature fruits from *Arabidopsis* (left) and *Capsella* (right) with tissues indicated as s (style), v (valve) and r (replum). Mediolateral and proximodistal orientations are indicated. (B) Fruit width plotted against length during *Capsella* (black diamonds) and *Arabidopsis* (grey crosses) fruit development (natural logarithm scales). The gradient for the fitted line for *Arabidopsis* (orange) is 0.49 (growth in length>width). There are two distinct gradients for *Capsella*: an early phase of 0.33 (green line, growth in length>width) and a later phase of 1.14 (blue line, growth rate in width>length). Red dashed line shows a gradient of 1 for comparison. (C) *Capsella* fruit length (black diamonds) and width (grey crosses) plotted against time from initiation to maturity. (D) *Arabidopsis* fruit length (black diamonds) and width (grey crosses) against time from initiation to maturity. In C and D the mean shapes of the fruit at each of the given stages are shown. Scale bars: 1 mm in C,D.

In common with most angiosperms, *Arabidopsis* fruits are derived from united carpels that encapsulate the developing seeds. The German author and philosopher Johann Wolfgang von Goethe proposed that all lateral plant organs are modifications of the same archetypal organ (von Goethe, 1790). In line with Goethe's hypothesis, carpels have been shown to have a leaf-like origin (Scutt et al., 2006). The lateral part of the *Arabidopsis* fruit develops into valves (the walls of the seed pod) that are fused to a medial replum. Between the valves and the replum, narrow strips of tissue made up of a few cell files form the valve margin where fruit dehisces to release the seeds upon maturity (Ferrándiz et al., 1999; Seymour et al., 2013). A style topped with stigmatic papillae develops at the apex of the fruits (Fig. 1A). The development and growth of the fruit are precisely coordinated across these diverse tissues to ensure the timely release of seeds upon maturity.

Some of the key regulators of fruit development in *Arabidopsis* have been identified and genetic interactions between them established. *FRUITFULL* (*FUL*) and *REPLUMLESS* (*RPL*) genes specify valve and replum formation, respectively, and they do so at least partly by restricting the expression of valve margin identity genes such as *SHATTERPROOF1* (*SHP1*), *SHP2*, *INDEHISCENT* (*IND*) and *ALCATRAZ* (*ALC*) (Liljegren et al., 2004; Dinneny et al., 2005).

Fruits from members of the *Capsella* genus have the same overall tissue composition as *Arabidopsis*, including two valves, a replum and style. However, the valves of mature *Capsella* fruits are extended at the distal end resulting in a heart-shaped appearance of the organ. In 1914, George Harrison Shull crossed the tetraploid *Capsella bursa-pastoris* (heart-shaped fruits) with a natural variant of *C. bursa-pastoris*, named ‘*heegeri*', which has cylindrical fruits. Shull found a 15:1 segregation in the F2 generation of heart to cylinder (Shull, 1914), leading him to suggest that two genetic loci contribute to the trait. This observation agrees with Sinnott's hypothesis two decades later that while certain genes will promote organ growth, others will be required to establish shape (Sinnott, 1935).

Here, we compare the formation of the heart-shaped fruit of *Capsella* with the cylindrical fruit of *Arabidopsis* to understand how organ shape is controlled and thus how the different fruit forms can emerge. Morphological and clonal analyses reveal patterns of anisotropic growth (when the ratio of growth rate in length to growth rate in width differs from 1) that can vary in both space and time between the species. We describe different phases during *Capsella* and *Arabidopsis* development, each including consecutive developmental stages (Roeder and Yanofsky, 2006). *Arabidopsis* shows similar levels of anisotropy during all phases of development, whereas *Capsella* shows a more complex pattern of anisotropy that changes from one phase to the next. Based on these findings we develop a model that accounts for both the resultant patterns of anisotropy and the fruit shape in each species. Given the conservation of tissue identities among Brassicaceae fruits, we also compare the role of the valve-identity factor FUL in fruit shape development and show that it exerts its effects during the late phase of growth. Our findings thus provide a framework for understanding the development of diverse fruit shapes within the Brassicaceae family.

## RESULTS

To determine when and how the shapes of *Arabidopsis* and *Capsella* diverge, we first compared the growth rates in overall length and width. We chose a starting point when the gynoecium primordia are similar in shape and size, ∼40 µm in length. This stage is referred to as 0 days after initiation (DAI) and is equivalent to stage 6 of *Arabidopsis* flower development (Roeder and Yanofsky, 2006). From 0-2 DAI defines an initial phase of high growth rate preferentially along the longitudinal axis of the gynoecium in both *Capsella* and *Arabidopsis* (Table 1). After this early phase of anisotropic growth, there is a drop in both growth rate in length and anisotropy in *Capsella* (Table 1, Fig. 1B,C), whereas in *Arabidopsis* anisotropy is maintained while growth rate in length is less reduced (Table 1, Fig. 1B,D). Thus, the development of *Capsella* and *Arabidopsis* fruits can be divided into two phases: an early phase of relatively high growth rate in length, followed by a second phase during which this growth rate is lower. Growth in width is constant during these two phases (Table 1).

**Table 1.**
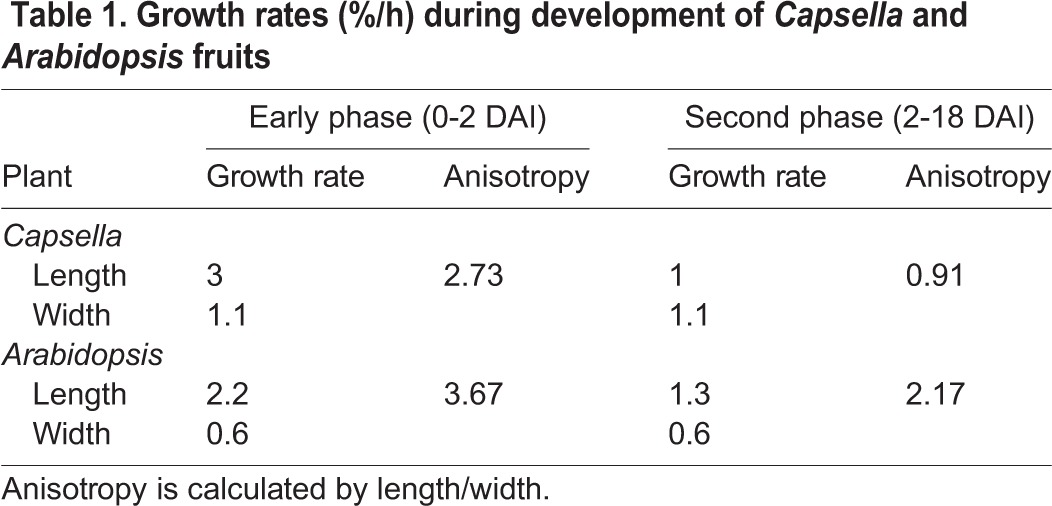
**Growth rates (%/h) during development of *Capsella* and *Arabidopsis* fruits**

**Fig. 2.**
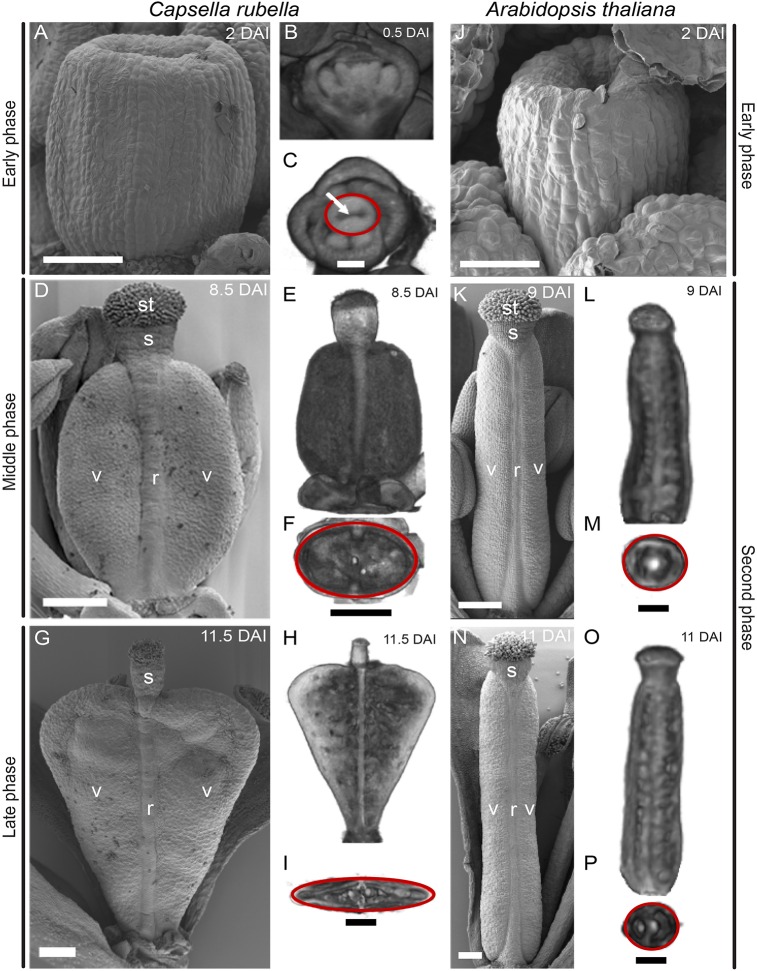
**SEM and OPT images of gynoecium shape development in *Capsella* and *Arabidopsis*.** (A-I) *Capsella rubella* gynoecium and fruit. (A) SEM of gynoecium at 2 DAI. (B,C) Virtual sections at 0.5 DAI in the longitudinal (B) and transverse (C) planes. A central groove is indicated (arrow). (D-F) As in A-C, but at 8.5 DAI. (G-I) As in A-C, but at 11.5 DAI. (J-P) *Arabidopsis thaliana* gynoecium and fruit. (J) SEM of gynoecium at 2 DAI. (K) SEM at 9 DAI. (L,M) Virtual sections at 9 DAI in the longitudinal (L) and transverse (M) planes. (N-P) As in K-M, but at 11 DAI. Shapes of gynoecium/fruit OPT cross-sections are indicated with red circles (C,F,I,M,P). The background in the OPT images in C,E,F,H,I,L,M,O,P was set to white using the software package VolViewer. st, stigma; s, style; v, valve; r, replum. Scale bars: 50 µm in A-C,J; 250 µm in D-I,K-P.

To determine the shape changes during these two phases we used optical projection tomography (OPT) and scanning electron micrographs (SEMs) to image different stages. In the early phase (0-2 DAI) the shapes of the *Capsella* and *Arabidopsis* gynoecia are similar: they initiate as an oval ridge with a central groove that grows into a hollow cylinder by 2 DAI (Fig. 2A-C,J). However, between 2 and 8.5 DAI the gynoecia of *Capsella* and *Arabidopsis* become morphologically distinct. The *Arabidopsis* gynoecium continues to elongate as a cylindrical shape throughout development (2-18 DAI), resulting in a long thin fruit (Fig. 2K,L,N,O) of near-circular circumference (Fig. 2M,P). By contrast, the *Capsella* gynoecium develops into a more rounded structure with an oval circumference and topped by a narrow style (8.5 DAI, Fig. 2D-F). This shape is known as an oblate spheroid (Hilbert and Cohn-Vossen, 1999). From 8.5 to 11.5 DAI, the base of the *Capsella* fruit becomes increasingly tapered and the distal part of the valves grows larger to produce a heart shape (Fig. 2G,H) that is flattened in cross-section (Fig. 2I). 9 DAI corresponds to the fertilisation stage (stage 13 of *Arabidopsis* flower development), and the characteristic heart shape of *Capsella* fruits therefore forms while the seeds develop inside. Based on these shape changes, the second growth phase in *Capsella* (Table 1) is divided into a middle (oblate spheroid) and late (heart-shape) phase, whereas in *Arabidopsis* the second phase shape is constant throughout development.

### Approach to measuring and modelling growth

**Fig. 3.**
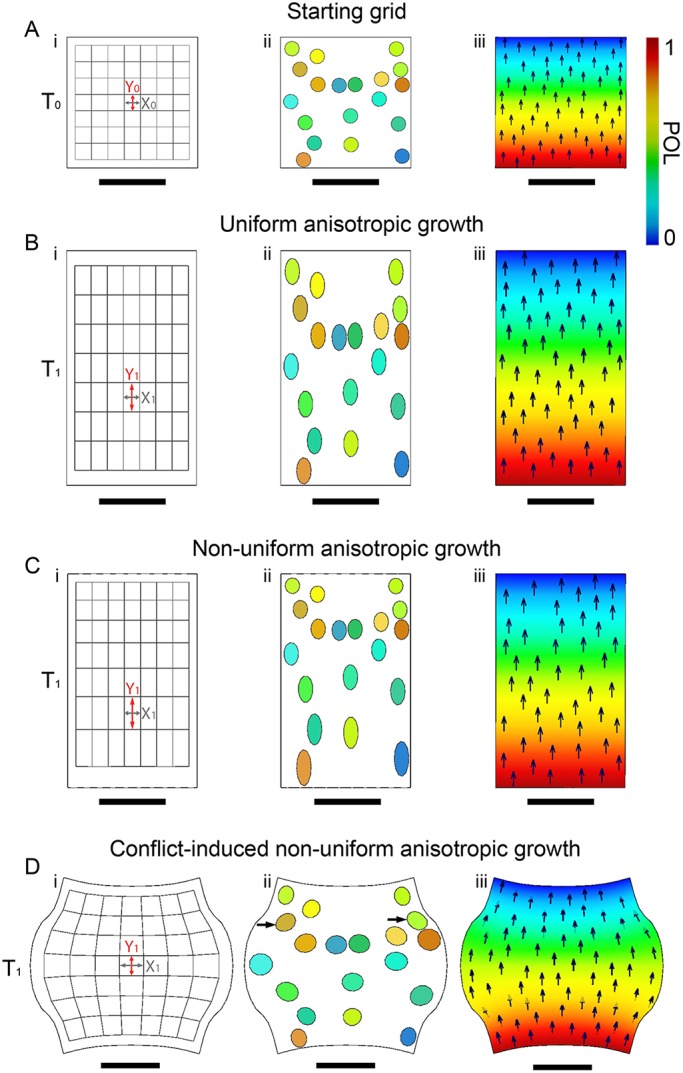
**Growing a 2D square.** (A) Starting 2D square at T_0_ marked with a grid (i), virtual clones (ii) and polarity field (iii). (B) Resultant rectangle shape after uniform anisotropic growth: grid has deformed uniformly (i) and virtual clones have uniform size and shape (ii). (C) Resultant rectangle shape as in B, but with non-uniform anisotropic growth (higher growth at the bottom). The grid has deformed non-uniformly (i) and the virtual clones differ in size and shape across the rectangle (ii). (D) With higher K_per_ in the middle than at the top and bottom, the shape will bulge in the middle and give rise to a rounded shape. This curvature was not specified but emerges as the result of tissue conflicts. Arrows (Dii) indicate clones that splay out at the edge of bulge. Scale bars: 1 mm.

The observed changes in fruit shape could be generated by many different growth patterns. Consider a simplified example of a 2D square piece of tissue that grows into a vertically elongated rectangle (Fig. 3). This transformation could be generated by a pattern of uniform anisotropic growth, in which growth rate along the vertical axis is higher than that along the horizontal axis. An initially square grid or set of marked circles would then be uniformly stretched (Fig. 3A,B). The vertical growth rate of each region (circle or square) would be:
(1)
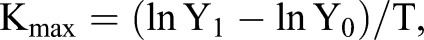
where T is time, Y_0_ is the length of the vertical axis at T_0_, and Y_1_ is the length of the vertical axis at T_1_, while the horizontal growth rate would be:
(2)
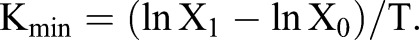
The square could also be transformed into an elongated rectangle by a pattern of non-uniform anisotropic growth, with higher growth rate at the bottom than the top (Fig. 3C). Sections in the original grid would then have different sizes in the resultant elongated rectangle. The growth rates of each region could again be estimated by taking the difference in the natural logarithms, although these values would no longer be uniform over the tissue.

Similar to marking the tissue with circles in the grid, individual cells could be marked in a biological system to capture the distribution of cellular growth patterns across a tissue. We used a Cre-Lox heat shock-inducible system, Brother of Brainbow (BOB) (Wachsman et al., 2011), to mark randomly distributed cells with either CFP or RFP upon induction. We marked cells at various times and imaged resultant clone patterns at specified lengths of the gynoecium: 300 µm, 500 µm, 1 mm, 2 mm and 4 mm, using florescence or confocal microscopy. We assumed an initial cell size of 7×7 µm based on measurements at 0 DAI in *Arabidopsis* and 4-6 DAI in *Capsella*. In *Capsella*, cell sizes were similar between 4 and 6 DAI and so it was assumed that cell size was maintained at 7 µm from 0 DAI (Table S1). As with the circles in the grid example (Fig. 3), assuming that the initial cells are of uniform size and isodiametric shape, growth rates along different axes can be estimated from the dimensions of the resultant clones.

The transformation of a square into a rectangle in 2D is more straightforward than the growth of a tissue, which can curve and deform in 3D as a result of mechanical constraints. In this situation, computational modelling is needed to relate the observed clonal patterns to the underlying specified growth patterns. We used the Growing Polarised Tissue (GPT) framework, in which growth rates can be specified by factors that are distributed across the tissue (Green et al., 2010; Kennaway et al., 2011; Kuchen et al., 2012; Sauret-Gueto et al., 2013). The tissue is modelled as a continuous sheet, termed the canvas, that is mechanically connected and can deform in 3D. A polarity field allows anisotropic growth to be incorporated. Polarity is specified with a diffusible factor, POLARISER (POL), which propagates from regions of high to low concentration (Fig. 3A-Diii). The gradient of POL is used to specify a local polarity, allowing parallel (K_par_) or perpendicular (K_per_) growth rates to be separately specified.

We distinguish between specified growth (inputs to the model) and resultant growth (output) (described by Kennaway et al., 2011). The specified growth rate of a region is the rate at which that region would grow if it were free from any mechanical constraints of surrounding tissue. The resultant growth rate is the rate at which that region grows when mechanical constraints of neighbouring tissue are taken into account. If the tissue has uniform isotropic growth rates, or all tissue grows with the same anisotropic growth rate in the same orientation (Fig. 3B,C), then there is no tissue conflict and specified and resultant growth are the same. In most other cases, conflicts between connected regions that are specified to grow at different rates or orientations generate deformations and curving of tissue (rotations) that are not explicit in the specified growth pattern but emerge through the mechanical conflicts. For example, if a square shape is grown with a higher K_per_ in the middle than at the top and bottom, the shape will bulge in the middle (Fig. 3D). The initial square grid has become curved and polarity is no longer uniformly vertical. The curvature (regional rotation) was not part of the specified growth but emerges as the result of tissue conflicts. If curvature does not fully resolve the conflicts, residual stresses are also generated. In our models, we assume that these residual stresses are dissipated during growth (Kennaway et al., 2011). Experimentally observed clone sizes and orientations do not give specified growth rates (inputs to the model) but can be compared with virtual clones or resultant growth rates generated as the result of running the model (outputs). For example, virtual clones (circles) in Fig. 3Dii splay out at the edge of the bulge (arrowed). This reorientation is not an explicit part of the initial specified growth pattern but emerges from the resultant growth and connectivity of the tissue.

**Fig. 4.**
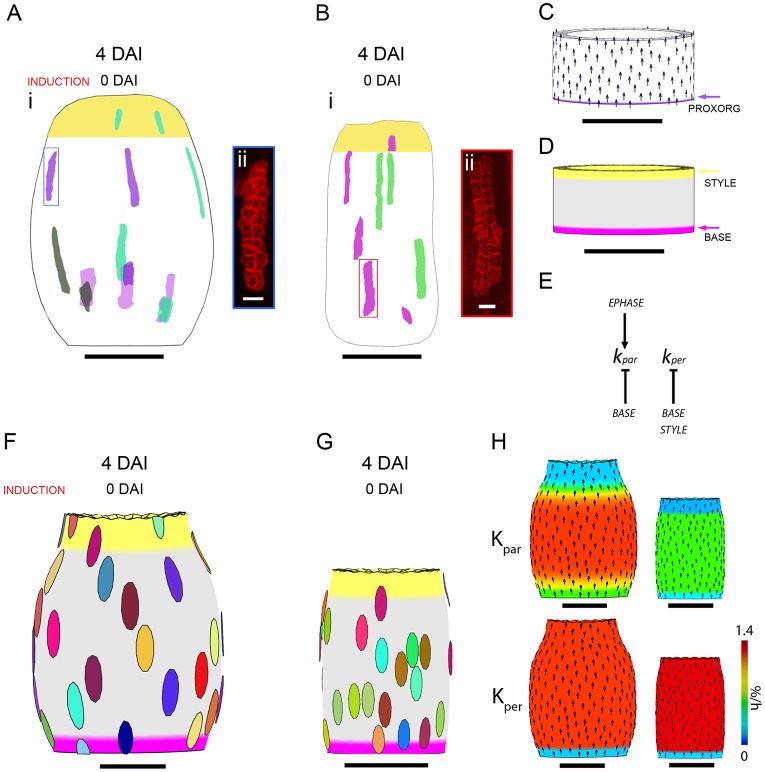
**Gynoecium clonal analysis and modelling of early phase and beginning of middle phase.** (Ai) Sector map with shapes and positions of clones induced at 0 DAI and imaged 4 days after induction in *Capsella* gynoecia. Clones were imaged from dissected gynoecia and warped onto an average gynoecium shape using a different colour for each gynoecium. (Aii) Image of a *Capsella* gynoecium epidermal clone (highlighted by a blue box in Ai). (Bi) Sector map with clones induced at 0 DAI and imaged 4 days after induction in *Arabidopsis* gynoecia. (Bii) Image of an *Arabidopsis* gynoecium epidermal clone (highlighted by a red box in Bi). (C) Canvas at 0 DAI for both *Capsella* and *Arabidopsis* models. The polarity field (arrows) depends on the production of a factor POL in the PROXORG region and degradation of POL everywhere at a constant rate. (D) Distribution of factors STYLE and BASE on the canvas at 0 DAI. (E) Growth regulatory network for both *Capsella* and *Arabidopsis* models. The basic rates of K_par_ and K_per_ differ in each model. BASE and STYLE are active during 3-4 DAI. (F) *Capsella* model outcome at 4 DAI showing virtual clone shapes and patterns. (G) *Arabidopsis* model outcome at 4 DAI showing virtual clone shapes and patterns. (H) K_par_ and K_per_ (colour key represents specified growth rates, %/h) with polarity field (arrows) plotted on *Capsella* (left) and *Arabidopsis* (right) models at 4 DAI. Scale bars: 10 µm in Aii,Bii; 50 µm in C,D; 100 µm in Ai,Bi,F-H.

Each model is composed of three components: a starting canvas shape (loosely based on the size and shape of the gynoecium at 0 DAI, simplified as a short oval cylinder) with regional factors, a growth regulatory network and a polarity system (Fig. 4C-E). The connectedness of the tissue results in shapes and features, such as curvature, that were not specified in the model and result from regional growth conflicts. Rather than match all the details of the clones and fruit shape changes, we tried to capture the general character of the clones and shape transitions in each phase.

### Early phase

The early phase is characterised by high growth rates in length and a cylindrically shaped gynoecium in both species. Because we were unable to easily image clones at 2 DAI, we imaged them at 4 DAI (300 µm length), which covers the early phase and beginning of the middle phase. We imaged epidermal clones for multiple samples and collated them onto a mean shape of the organ at 4 DAI to generate a clone map (Fig. 4). The mean shapes of the *Capsella* and *Arabidopsis* gynoecia have already started to diverge at 4 DAI: *Capsella* has a wider and more rounded shape (Fig. 4Ai) than *Arabidopsis* (Fig. 4Bi). This difference in shape is most likely generated in the middle phase (see below).

Consistent with length/width (L/W) growth rates, the clones that capture the early phase of growth (0-2 DAI) are elongated along the longitudinal axis in both *Capsella* and *Arabidopsis* (Fig. 4Aii,Bii). Comparing *Capsella* and *Arabidopsis* clones induced at 0 DAI and imaged at 4 days after induction gave an average L/W ratio of 5.2 and 7.7, respectively. This anisotropic shape of the clones is correlated with cell division rates, with two to three rounds of cell division along the longitudinal axis and zero or one round of cell division along the circumferential axis in both species. Growth is mostly uniform across the gynoecium in both species. However, clones near the base and the apex in *Capsella* and *Arabidopsis* are shorter, suggesting slower growth along the proximal distal axis in these regions (Fig. 4Ai,Bi).

To determine how the growth rate of the clones compares to whole-organ growth values, the average growth rates of the clone major axis (K_max_) and minor axis (K_min_) were calculated (Table 2) based on growth rates in defined regions (Table S3). Since the clones are anisotropic along the longitudinal axis of the gynoecium, K_max_ is aligned with the longitudinal axis of the gynoecium and K_min_ is aligned with the circumference. The values of K_max_ are comparable to high growth rates in length for the early and middle phases in both *Arabidopsis* and *Capsella* (Table 2). However, the values of K_min_ are lower than the whole-organ growth rates in width in both species (Table 2). This could be explained if the cells were not isodiametric in shape at the time of induction. For example, if the cells were smaller than 7 µm in width, growth rates would be underestimated. Overall, the clone patterns show that the high longitudinal growth in the early stages of development is distributed throughout the gynoecium of both species and slightly lower towards the base and style.

**Table 2.**
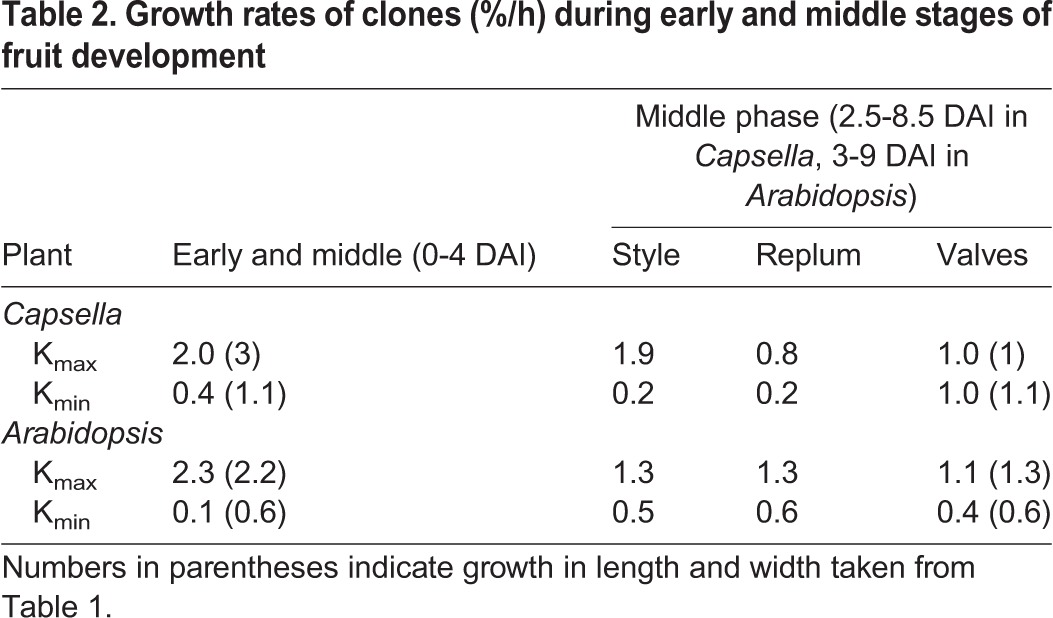
**Growth** **rates of clones (%/h) during early and middle stages of fruit development**

To model this anisotropic growth during the early phase using the GPT framework, we created a longitudinal gradient of POL in the initial canvas. This was achieved by having a region of POL production at the base. The result is a proximodistally oriented polarity field (Fig. 4C). To account for the observed anisotropy in growth, the specified growth rates are higher parallel to the polarity than perpendicular to the polarity (for *Capsella* K_par_=1.4%/h and K_per_=1.15%/h and for *Arabidopsis* K_par_=1.3%/h and K_per_=0.6%/h). These growth rates were specified based on L/W growth rate ratios during the individual growth phases of the gynoecium and fruit. To enhance longitudinal growth during the early phase, we introduced a factor (EPHASE) that promotes K_par_ from 0-2 DAI (Fig. 4E). As the clonal analysis spans 0-4 DAI, EPHASE is inactive during the 3-4 DAI period. In addition, the fact that both species only have a few clones near the base and presumptive style suggests lower growth in these regions, and so we introduced two additional factors active during 3-4 DAI: BASE, which inhibits K_par_ and K_per_; and STYLE, which inhibits K_per_ (Fig. 4D,E). Growth specified in this way causes the canvas to deform to give resultant sizes and shapes that broadly match those of the gynoecia observed at 4 DAI (Fig. 4F,G). The output shape for both models tapers towards the base and style because of the inhibition of K_per_ in these regions, which creates a conflict in growth rate between these regions and the adjoining valve (Fig. 4H). The *Capsella* shape tapers more than the *Arabidopsis* shape because of the higher rate of K_per_.

To determine whether the model could account for the experimental pattern of clones, virtual clones (circular regions) were introduced into the models at 0 DAI. The resultant virtual clone shapes (Fig. 4F,G) are broadly similar to the experimental clones (Fig. 4Ai,Bi): they are anisotropic in shape, elongated parallel to the proximodistal axis and are slightly wider in the model for *Capsella* compared with *Arabidopsis*. In addition, the growth rates and anisotropy of clones in the model are similar to those of experimental clones (Table S2). Thus, a proximodistal polarity field combined with a set of growth rules that are similar between the species can explain both gynoecium forms during the early phase and early part of the middle phase.

### Middle phase

**Fig. 5.**
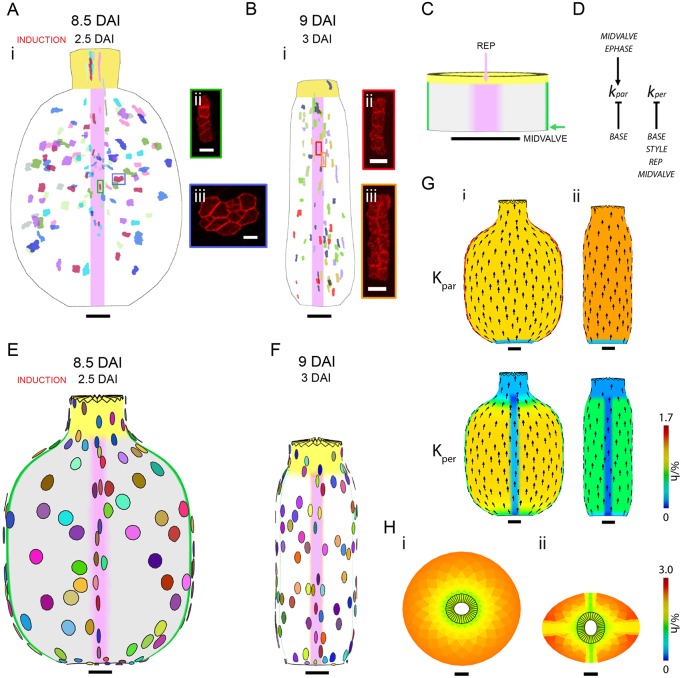
**Gynoecium clonal analysis and modelling of the middle phase.** (Ai) Sector map with clones induced at 2.5 DAI and imaged 6 days after induction in *Capsella* gynoecium. (Aii,Aiii) Images of *Capsella* gynoecium epidermal clones in replum (green box in Ai) and valve (blue box in Ai), respectively. (Bi) Sector map with shapes and positions of clones induced at 3 DAI and imaged 6 days after induction in *Arabidopsis* gynoecium. (Bii,Biii) Images of *Arabidopsis* epidermal clones in replum (red box in Bi) and valve (orange box in Bi), respectively. (C) Canvas at 0 DAI for both *Arabidopsis* and *Capsella* models showing the distribution of factors REP and MIDVALVE. (D) Growth regulatory network for both *Capsella* and *Arabidopsis* models covering early and middle phase. All factors except for EPHASE are specific to the middle phase. The basic rates of K_par_ and K_per_ differ in each model. (E) *Capsella* model outcome at 8.5 DAI showing virtual clone shapes and patterns. (F) *Arabidopsis* model outcome at 9 DAI showing virtual clone shapes and patterns. (G) Specified K_par_ and K_per_ (colours) with polarity field mapped onto *Capsella* canvas at 8.5 DAI (i) and *Arabidopsis* canvas at 9 DAI (ii). (H) Cross-sections of models with K_per_ inhibited only in style and base (i) and with K_per_ inhibited in style, base, replum and midvalve as well as promotion of K_par_ in midvalve (ii). Coloured chart represents resultant growth rates (%/h). Scale bars: 10 µm in Aii,iii,Bii,iii; 50 µm in C; 100 µm in Ai,Bi,E-H.

The middle phase is characterised by a reduced growth rate in length compared with the early phase (Table 2). During the middle phase, *Capsella* produces a rounded gynoecium with a flattened cross-section and *Arabidopsis* produces a longer and cylindrically shaped gynoecium. To capture the growth patterns that generate this divergence of shape, clones were induced at 2.5-3 DAI and imaged 6 days later when the gynoecia reached ∼1 mm in length (Fig. 5A,B). At this stage, distinct regions and tissue types become evident at the fruit surface in both species. As with other members of the Brassicaceae family, *Arabidopsis* and *Capsella* fruits develop lateral valves that are attached to a medial replum and the structure is topped with a style and stigmatic tissue (Fig. 2) (Roeder and Yanofsky, 2006). To simplify the analysis, we considered clones induced in the style, replum and valve tissues individually.

Clones induced in the style of both species are elongated along the proximodistal axis (Fig. 5Ai,Bi). This is captured by calculating the average L/W ratio of the clones induced in the style in *Capsella* as 8.4 (±0.04 s.d., *N*=2) and 3.1 (±0.2, *N*=5) in *Arabidopsis* (Table S3). This anisotropy is correlated with two to three rounds of cell division along the major axis and no cell division along the minor axis.

Similar to the clones in the style, clones induced in the replum in both species exhibit clear elongation along the proximodistal axis (Fig. 5Aii,Bii). The average L/W ratio of the clones induced in the replum is 2.8 (±1.0, *N*=21) for *Capsella* and 5.7 (±2.0, *N*=5) for *Arabidopsis* (Table S3). The anisotropic shape of the clones is correlated with higher cell division rates along the major axis of the clones (two to three rounds) compared with the minor axis (zero or one round). Overall, during the middle phase in both *Capsella* and *Arabidopsis* the growth in the style and the replum is anisotropic, with a higher growth rate along the proximodistal axis.

Unlike clones in the style and the replum, clones induced in the valves show a divergent pattern of growth during the middle phase in *Capsella* and *Arabidopsis*. In *Capsella*, these valve clones are uniform in size and mostly isotropic in shape (Fig. 5Ai,iii). In *Arabidopsis*, valve clones are also uniform in size but elongated along the proximodistal axis of the gynoecium (Fig. 5Bi,iii). The average L/W ratios of the clones are 1.0 (±0.4, *N*=22) in *Capsella* and 3.0 (±0.9, *N*=83) in *Arabidopsis* (Table S3). This is correlated with differences in cell division patterns, as in *Capsella* there are one to two rounds of cell division along both axes (Fig. 5Aiii), whereas in *Arabidopsis* there are two to three rounds of cell division along the proximodistal axis and one round of cell division along the mediolateral axis (Fig. 5Biii). The anisotropic shape of the clones is also correlated with differences in cell expansion, as valve cells are no longer isodiametric but wider in *Capsella* (Fig. 5Aiii) and longer in *Arabidopsis* (Fig. 5Biii).

To determine how growth rates vary between regions during the middle phase, the growth rates of clones in the style, replum and valve regions were calculated (Table 2). For *Capsella*, K_max_ is similar for valves and replum but higher in the style, whereas for *Arabidopsis* K_max_ is highest in the replum (Table 2). By contrast, K_min_ in *Capsella* (along the circumferential axis) is lower in the replum and style than in the valves (Table 2), whereas K_min_ in *Arabidopsis* is similar across all three tissues. The growth rates in the valves of both species match the whole-organ L/W growth rates (Table 2).

When calculating the growth rates using the major and minor axes of the clones in valves of *Capsella*, the orientations of the clones did not always align parallel to the proximodistal axis of the gynoecium. Instead, the clones in the valves diverge away from the base and converge back towards the style (Fig. S1A). By contrast, in *Arabidopsis* valves the major axes of the clones align with the proximodistal axis of the gynoecium (Fig. 5Bi). Thus, clone patterns in the valves of *Capsella* show uniform isotropic growth with a divergent then convergent pattern, whereas for *Arabidopsis* they show uniform anisotropic growth parallel to the proximodistal axis.

Models of the middle phase need to account for the oblate spheroid shape of *Capsella* and the cylindrical shape of *Arabidopsis* gynoecium by 8-9 DAI. They must account for the proximodistal anisotropic clone patterns in the style and the replum in both species, and for the valve clones, which are wider with a divergent and convergent pattern in *Capsella*, as opposed to the narrow, parallel clone patterns in *Arabidopsis*.

These gynoecium shapes and clone patterns cannot be accounted for by continuing with the simple growth interactions established in the early phase (0-2 DAI, Fig. S2). To account for the clone patterns and growth rates during the middle phase we inactivated general promotion of Kpar (EPHASE is switched off). In addition to STYLE and BASE, we introduced a factor, REP, into the model. REP is expressed in a central band and inhibits K_per_ (Fig. 5C,D). This interaction was proposed to account for clones in the replum being highly anisotropic along the proximodistal axis (Fig. 5Aii,Bii). The canvas was grown to 8.5 DAI for *Capsella* and 9 DAI for *Arabidopsis.* The longitudinal views of the modelling outputs broadly match the observed gynoecium sizes and shapes at a similar stage (Fig. 5E,F). In addition, the virtual clones in the style and replum regions resemble the anisotropic shape of the observed clones in these regions. The growth rates and anisotropy of the virtual clones are similar to those observed in the middle phase (Table S3). In both models, the shape tapers at the base and style because of inhibition of K_per_ in these regions, although this is more extreme for *Capsella*. This differential growth creates a conflict that results in deformation of the polarity field so that it diverges from the base and converges towards the style, accounting for the virtual clones in the valve showing a similar pattern (Fig. 5Ai,E,Gi, Fig. S2). Virtual clones in the *Arabidopsis* model also run parallel to the polarity, which is parallel to the proximodistal axis of the model, matching the observed clones (Fig. 5Bi,F,Gii). Therefore, to account for the gynoecium shape changes and observed clone patterns during the middle phase it is sufficient to reduce the rate of K_per_ in the style, base and replum regions, while continuing with higher value of K_per_ in *Capsella* compared with *Arabidopsis* established during the early phase.

These regional growth interactions can account for the observed clone patterns and many of the gynoecium shape features by the end of the middle phase. However, a major difference between the model and experimental observations is evident from cross-sections of *Capsella* gynoecia, which reveal a flattened oval that contrasts with the rounded shape obtained with the model (Fig. 2F, Fig. 5Hi). This reflects the gynoecium having an oblate spheroid rather than a spherical shape. Given the oblate spheroid shape of the valve, the distance from the style to the base is greater when following the middle of the valve than when following the replum (Fig. S3). We therefore hypothesised that enhanced growth along the midvalve might be needed to account for this shape. To test this idea, we introduced a factor, MIDVALVE, into the *Capsella* model. MIDVALVE is active in a thin band down the middle of the valves (Fig. 5C). This observation was included in the model such that MIDVALVE promotes K_par_ giving rise to narrower sectors close to the midvalve (Fig. 5E). To stop the midvalve from becoming a wide region, K_per_ was inhibited by MIDVALVE. Higher K_par_ in the midvalve creates a conflict with the rest of the valve that tends to flatten the shape. The resultant cross-sectional shape of the *Capsella* model at 11.5 DAI broadly matches the flattened oval shape of the gynoecium at this stage (Fig. 5Hii).

Taken together, this analysis shows that the observed shape changes and clone patterns during the middle phase can be accounted for using regional factors in the midvalve, style and replum to alter local specified growth rates parallel or perpendicular to an initial proximodistal polarity.

### Late phase

**Fig. 6.**
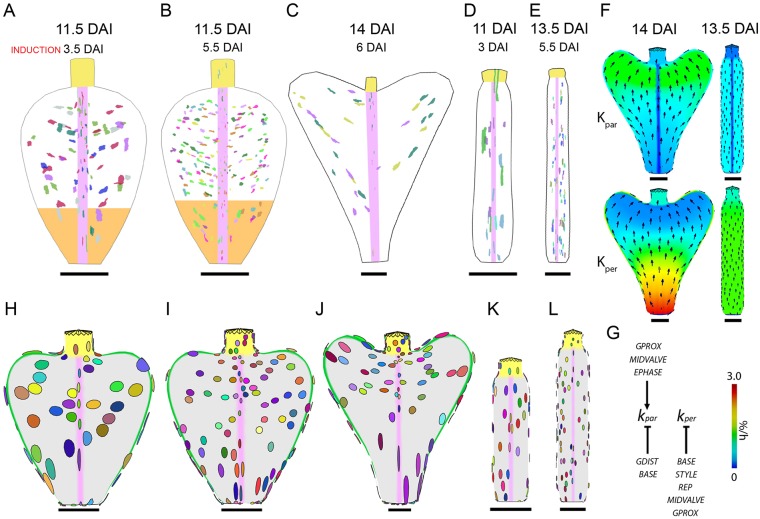
**Clonal analysis and modelling the late phase.** (A-C) Sector map of *Capsella* fruit with clones induced at (A) 3.5 DAI and imaged 6 days after induction, (B) 5.5 DAI and imaged 6 days after induction and (C) 6 DAI and imaged 8 days after induction. Orange area in A and B indicates a region near the base that is enriched for clones that are elongated along the proximodistal axis. (D,E) Sector maps of *Arabidopsis* fruit with clones induced at (D) 3 DAI and (E) 5.5 DAI and imaged 8 days after induction. (F) Specified K_par_ and K_per_ (colours) with polarity field mapped onto *Capsella* canvas at 14 DAI (left) and *Arabidopsis* canvas at 13.5 DAI (right). (G) Growth regulatory network for the *Capsella* model. Factors GDIST and GPROX are only active in the late phase. GMIDVALVE plays a similar role to MIDVALVE in promoting Kpar in the late phase. (H,I) *Capsella* model outcome at 11.5 DAI. Virtual clones were induced at 3.5 DAI and 5.5 DAI, respectively. (J) *Capsella* model outcome at 14 DAI. Virtual clones were induced at 6 DAI. (K) *Arabidopsis* model outcome at 11 DAI. Virtual clones were induced at 3 DAI. (L) *Arabidopsis* model outcome at 13.5 DAI. Virtual clones were induced at 5.5 DAI. Coloured chart represents resultant growth rates (%/h). Scale bars: 250 µm in A,B,D,H,I,K; 500 µm in C,E,F,J,L.

The late phase is characterised by a transformation in *Capsella* from an oblate spheroid to a heart-shaped fruit. In *Arabidopsis*, no major change in shape is observed during the late phase. However, in both species overall growth rates in length and width are maintained at similar rates to the middle phase. To capture the growth patterns in *Capsella* during the late phase we induced clones at 3.5, 5.5 and 6 DAI (middle phase) and imaged 6 or 8 days after induction when the fruit length had reached 2 mm (Fig. 6A,B) and 4 mm (Fig. 6C), respectively. Similarly, in *Arabidopsis* we induced clones at 3 and 5.5 DAI and imaged 8 days after induction when the fruit length had reached 2 or 4 mm. The clones reflect growth at the end of the middle phase and the late phase and, at the time of induction, cell shapes could not be assumed to be isodiametric. For this reason, we did not analyse growth rates of clones during the late phase but focussed on clone shapes and distributions.

The clone patterns in the style and replum are similar throughout the middle and late phases, being elongated parallel to the longitudinal axis of the fruit in both species. However, clone patterns in the valves in *Capsella* differ between the middle and late phase. Whereas middle phase clones are nearly isotropic (Fig. 5Aiii), clones that grew during the late phase are highly anisotropic (Fig. 6B,C). The orientation of late phase clones varies across the valves in *Capsella*, with clones near the base being elongated more along the longitudinal axis but clones in the middle and distal regions being elongated more diagonally (most clearly observed in Fig. 6C). The clone patterns during the late phase therefore reveal a dynamic redistribution of anisotropic growth in the *Capsella* valves as the fruit undergoes the transition from oblate spheroid to a heart-shaped structure. By contrast, clone shapes and orientations in the valves of *Arabidopsis* remained similar between the middle and late phases (Fig. 5Biii, Fig. 6D,E).

A model of the late phase in *Capsella* needs to account for the shape change of the fruit and for the anisotropy and orientation of the clones. Continuing to grow the *Capsella* model to later stages with the same interactions as discussed above cannot account for these shape changes and clone patterns (Fig. S4). By contrast, when the *Arabidopsis* canvas continues to grow based on the interactions detailed in the middle phase, the resultant shapes and clone patterns resemble the observed fruit shapes and clone patterns of the same stages (Fig. 6D,E,K,L). Additional growth interactions are therefore needed in the *Capsella* model to generate the heart shape, whereas the *Arabidopsis* model can remain the same in the middle and late phases.

The clone patterns suggest that the additional growth interactions needed in the *Capsella* fruit during the late phase differ for the proximal and distal regions. To distinguish the proximal region and the distal region we introduced two factors, GPROX and GDIST, which are distributed in opposing linear gradients in the valves (Fig. S5). These factors only influence growth rates after 8 DAI. Clones near the base are elongated more along the longitudinal axis, suggesting more growth parallel to the polarity. Therefore, GPROX promotes K_par_ and inhibits K_per_ (Fig. 6G). In the distal region, the polarity field curves to converge on the style (Fig. 6F) and clones are elongated perpendicular to this polarity. We therefore postulated that GDIST inhibits K_par_ (Fig. 6G). These specified growth patterns create a conflict between the proximal region with relatively low K_per_ (and high K_par_) and the distal region with relatively high K_per_ (and low K_par_). A factor GMIDVALVE was introduced as a linear gradient in the midvalve region (Fig. S5). Similar to MIDVALVE in the middle phase, GMIDVALVE promotes K_par_ to maintain the flattened cross-sectional shape. In addition, a factor APEX was introduced that inhibits K_par_ specifically in the midvalve at the apex of the shoulders to prevent isotropic growth in the shoulder region (Fig. S5). The resultant shapes generated by the model are similar to those of the *Capsella* fruit at equivalent stages (Fig. 6A-C,H-J). Consistent with the experimental data, virtual clones are elongated longitudinally in the proximal region and diagonally in the distal and middle regions (Fig. 6H). As the canvas grows the virtual clones diverge increasingly towards the shoulders of the valve (Fig. 6J). Overall, the model postulates a change in specified growth rates after the middle phase, with K_par_ promoted near the base and K_per_ promoted in the distal regions, and this can account for the final fruit form and the clone patterns of the *Capsella* fruit during the late phase.

### FRUITFULL modifies growth during the late phase

**Fig. 7.**
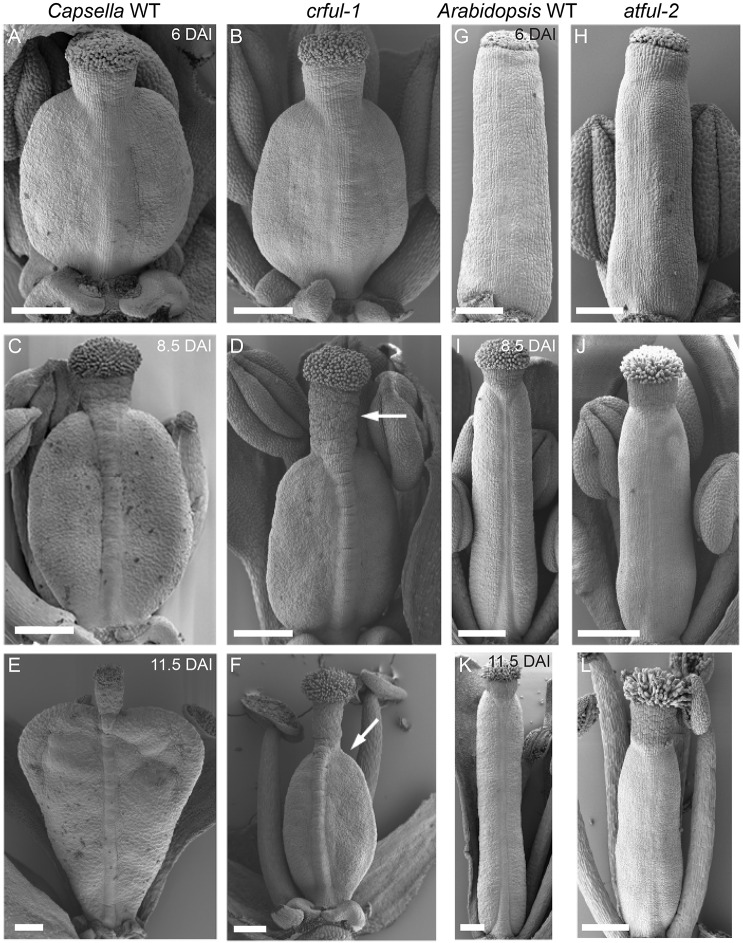
***CrFUL* is necessary for the late phase growth in *Capsella* and *Arabidopsis*.** (A,C,E) *Capsella* wild-type gynoecium at 6, 8.5 and 11.5 DAI, respectively. (B,D,F) *crful-1* at corresponding stages to wild type based on morphological features of the flower. Arrows indicate elongated style compared with wild type (D) or absence of the distal tips in *crful-1* fruit (F). (G,I,K) *Arabidopsis* wild-type gynoecium at 6, 8.5 and 11.5 DAI, respectively. (H,J,L) *atful-2* at corresponding stages to wild type. Scale bars: 100 µm in A,B,G,H; 250 µm in C-F,I-L.

In *Capsella* during the late phase, growth patterns change from the middle phase to transform an oblate spheroid into a heart-shaped fruit. The model predicts that genetic factors active after ∼8 DAI will be important for generating the heart form. A gene known to be important at the later stages of fruit development in *Arabidopsis* is *FRUITFULL* (*AtFUL*), which encodes a member of the MADS-box transcription factor family (Gu et al., 1998). Mutations in *AtFUL* affect fruit growth, as valve cells fail to elongate after fertilisation (Ferrándiz et al., 2000). We identified a likely *Capsella FUL* orthologue (*CrFUL*) in the genome sequence of the diploid *C. rubella* (Slotte et al., 2013). To test whether this gene has a similar effect on growth at late stages of *Capsella* fruit development as observed in *Arabidopsis*, we set out to identify *ful* mutant alleles in *Capsella*. We generated a mutant population in *C. rubella* by EMS mutagenesis. This population formed the basis for both a forward genetic screen and a targeted induced local lesions in genomes (TILLING) platform for reverse genetics. We screened mutant lines for fruit shape deformities and identified two mutants by forward genetic screening with a *ful*-like fruit phenotype. Mutations in the *CrFUL* gene in these two individuals were confirmed by sequencing and the effect of these mutations on the phenotype was confirmed by lack of complementation in F1 plants originating from crosses between the alleles (Fig. S6). These two alleles were named *crful-1* and *crful-2*. A third allele, *crful-3* was identified by TILLING and displayed a partial phenotype (Fig. S6). As in wild type, the gynoecium of *crful-1* develops with an oblate spheroid shape before fertilisation, but with a longer style than wild-type gynoecia (Fig. 7A-D). After fertilisation, when the shoulders develop in wild-type fruits, the *crful-1* fruits remain rounded (Fig. 7E,F). This rounded shape becomes slightly elongated but is retained at all subsequent stages of fruit development. This is consistent with the phenotype of *ful* mutant alleles in *Arabidopsis*, where gynoecia are largely indistinguishable from wild type until fertilisation (Fig. 7G-J), and the phenotype only becomes clearly evident during post-fertilisation fruit growth (Fig. 7K,L). These data show that *CrFUL* influences *Capsella* fruit shape mainly by modulating growth during the late phase.

**Fig. 8.**
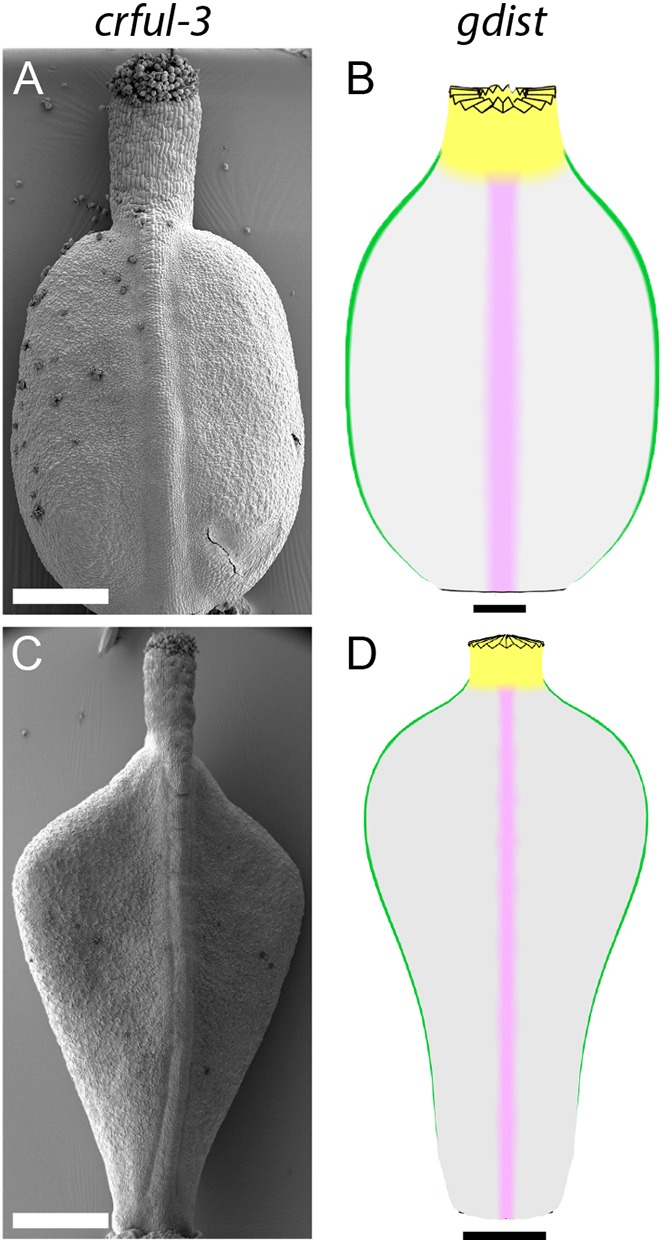
**CrFUL has overlapping activities with GDIST.** (A,C) SEM images of *crful-3* fruit before and after fertilisation. (B,D) *Capsella* model with GDIST removed (*gdist*) at corresponding stages. Models are shown for 8 DAI (B) and 14 DAI (D). Scale bars: 100 µm in B; 200 µm in A; 500 µm in C,D.

To test which factors in the model could represent the activities of *FUL*, we compared the phenotype of a weak *crful* mutant allele (*crful-3*) at two developmental stages with a model in which the activity of GDIST is removed and overall growth (K_par_ and K_per_) reduced (*gdist* mutant) (Fig. 8). Similar to the *crful-1* allele, *crful-3* gynoecium forms an oblate spheroid shape before fertilisation (Fig. 8A, Fig. S6), which is also observed at the end of the middle phase in *gdist* (Fig. 8B). After fertilisation, the *crful-3* fruit, like the wild type, develops a tapered base, but fails to generate the shoulders of the heart (Fig. 8C). The shape of *gdist* in late phase broadly matches that of the *crful-3* fruit (Fig. 8D). However, one difference between *crful-3* and *gdist* is the longer style of *crful-3*, which cannot be accounted for by simply removing GDIST from the model. This experiment suggests that GDIST and promotion of overall growth represents at least some of the activities of *FUL* during the late phase.

### Varying regional growth rates can explain much of the fruit shape diversity in the Brassicaceae

To test if this model of fruit development can account for other fruit shapes in the Brassicaceae we simplified the model to consider each subregion in turn. Consider the simplest growth pattern: isotropic growth of a cylinder with reduced K_per_ in the style and base gives rise to a spherical form (Fig. S2). Spherical fruits are present in the Brassicaceae, such as *Neslia paniculata* (Francis and Warwick, 2003), broadly matching the characteristics of this simple model.

**Fig. 9.**
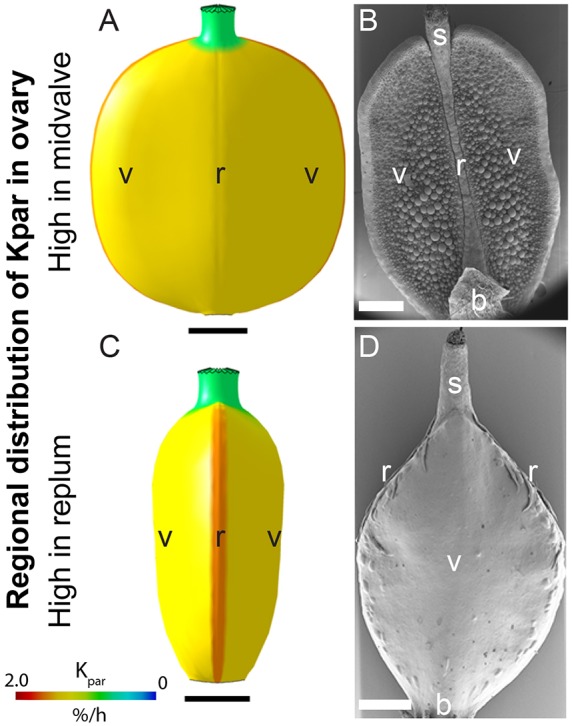
**Changes to the model can explain the shape of fruits from other Brassicaceae species.** (A,C) Models showing oblate spheroid-shaped fruits obtained via promotion of K_par_ in the midvalve (A) and replum (C) regions. View is facing the replum. Value of K_par_ is indicated by the colour key. (B,D) SEM images of mature fruit from *Lepidium campestre* (B) and *Alyssum maritimum* (D). The view in B is facing the replum, while that in D is facing the midvalve. Replum (r), valves (v), style (s) and base (b) are indicated. Scale bars: 500 µm.

Another common fruit shape observed in the Brassicaceae is the oblate spheroid form, similar to the gynoecium of *Capsella* before fertilisation. This spheroid form can be flattened either laterally, as for mature fruits from *Lepidium campestre*, or medially, as for *Alyssum maritimum* mature fruits. We have already shown that when K_par_ is relatively high in the midvalve (in *Capsella*) the model is flattened laterally (Fig. 5Hii). We found that if we continued to grow the model with MPHASE rather than introducing LPHASE, the model broadly accounts for the shape of laterally flattened spheroid fruit such as in *Ledipium* (Fig. 9A,B, Fig. S3). To test whether the replum could also be important for controlling fruit flatness, we promoted K_par_ by REP. The resulting form is flattened medially (Fig. 9C), with the replum tissue around the rim of a flattened spheroid, similar to the mature fruit of *Alyssum* (Fig. 9D). Therefore, relative rates of K_par_ in the midvalve or replum are important for controlling the flatness of the model to generate a spheroid shape. Overall, varying the model framework developed for *Arabidopsis* and *Capsella* fruits can account for at least basic fruit shape diversity in the Brassicaceae.

## DISCUSSION

The wide variation in organ morphology among otherwise closely related species makes the Brassicaceae family an excellent subject for comparing mechanisms underlying organ shape formation (Hay and Tsiantis, 2006). In this study, we combine experimental analysis of growth patterns with computational modelling to understand how dynamic changes in specified growth patterns can lead to the different shapes of fruits from *A. thaliana* (cylindrical fruits) and *C. rubella* (heart-shaped fruits). Our analysis covers specific stages of fruit development, and both qualitative and quantitative observations from these experiments were used to evaluate models for growth at each stage and for both types of fruit.

The models presented here make six key predictions that can explain the difference in fruit shapes observed in the Brassicaceae and can be further tested experimentally. (1) A common proximodistal polarity field is established early and deforms with the tissue (Fig. S7). (2) Different patterns of anisotropic growth are oriented by the polarity field, with growth rates being specified as parallel (K_par_) or perpendicular (K_per_) to the local polarity. (3) Anisotropic specified growth patterns switch at different growth phases: early, middle and late. (4) The tapered shape is due to relatively low K_per_ in the base and style in both models, but is more extreme for *Capsella*. (5) Relative specified growth rates in the narrow vertical regions within the fruit (midvalve or replum) are important for creating tissue conflicts that generate the cross-sectional shape. When K_par_ is promoted in these regions the shape is flattened, generating an oblate spheroid shape. In the case where K_par_ is promoted in the midvalve (like *Capsella*) the shape is flattened laterally (Fig. 5), whereas if K_par_ is promoted in the replum the shape is flattened medially (Fig. 9). (6) The heart shape of *Capsella* is formed after the oblate spheroid has been established, where the polarity field in the valve converges towards the style and provides a potential angle for the shoulders of the fruit to form. Together with a redistribution of K_par_ and K_per_ in a graded pattern in the valve, these conflicts can generate a heart shape, as observed in *Capsella*.

In this model of *Capsella*, the divergent/convergent pattern of growth is explained by a proximodistal polarity field that deforms with the growing tissue (Fig. S7). This type of polarity organisation is similar to a model of *Arabidopsis* leaf development in which a proximodistal polarity field deforms during growth leading to a divergent polarity field at the base and then convergent field at the tip (Kuchen et al., 2012). The same type of polarity organisation can also explain the parallel growth patterns of the *Arabidopsis* fruit. This common feature of polarity organisation between leaves and carpels might be a result of the evolutionary origin of the carpel from two fused leaves (Scutt et al., 2006).

A key question is how genes control the factors in the model. Several candidate genes may be involved in specifying the spatiotemporal pattern of factors in the models of *Arabidopsis* and *Capsella*. Some known genes have similar expression patterns and could carry out the roles of the hypothesised factors in the models. For example, genes encoding NGATHA and STYLISH transcription factors are expressed in the distal region from the earliest stages of gynoecium initiation (Kuusk et al., 2006; Trigueros et al., 2009; Alvarez et al., 2009). They act redundantly, and when all are knocked out the style does not develop. Similarly, expression of the replum identity factors *REPLUMLESS*, *BREVIPEDICELLUS* and *WOX13* is tightly constrained to the replum (Roeder et al., 2003; Alonso-Cantabrana et al., 2007; Romera-Branchat et al., 2012). We are not aware of any genes that are specifically expressed or inhibited in the midvalve region of the *Arabidopsis* fruit. However, development of the main vein in this region sets it apart from the rest of the valve. Gradients of factors such as GPROX and GDIST exist in the *Arabidopsis* fruit. These include *miRNA156* and members of the *SPL* family, which – when misexpressed – can alter fruit shape (Xing et al., 2013). It is therefore possible that genes known to regulate fruit development control factors in the model.

The shape changes of the *Capsella* gynoecium and fruit are regulated in developmental time. First, there is a transition from early to middle phase, giving rise to the oblate spheroid shape. Second, a transition occurs upon fertilisation from middle to late phase, leading to formation of the heart shape. Time-varying growth patterns have also been proposed for the *Arabidopsis* leaf and petal, where basic patterns of growth are established early on but are then gradually modulated over time (Kuchen et al., 2012). Similarly, a model for the complex *Antirrhinum* flower postulates a switch in growth patterns during development (Green et al., 2010).

A candidate gene for modulating gynoecium growth in time is *FUL*, which has been shown to be essential for proper fruit development in several angiosperms (Gu et al., 1998; Pabon-Mora et al., 2012; Wang et al., 2014). *AtFUL* expression is restricted to the valves of the *Arabidopsis* fruit, where it promotes valve identity and expansion after fertilisation (Ferrándiz et al., 2000). In terms of the model presented here, *AtFUL* may be needed for promotion of growth parallel to the polarity (K_par_) during the late phase in *Arabidopsis*. The *CrFUL* gene in *Capsella* also mainly affects growth in the late phase, as the *crful-1* mutant attains the oblate spheroid shape during middle phase but fails to form the heart shape after fertilisation. Interestingly, there is a strong resemblance between fruits from the *crful-1* mutant identified here and the *C. bursa-pastoris* ‘*heegeri*' variant (Shull, 1914), suggesting that it might be the two *CrFUL* genes of the tetraploid *C. bursa-pastoris* that are mutated in ‘*heegeri*'. *CrFUL* may be required to promote differential growth, leading to the heart shape. In agreement with this, the shape of *crful-3* fruit broadly matches a model in which GDIST, which inhibits K_par_ at the distal end, is removed and overall growth reduced. K_per_ is reduced at the distal end (by removing GDIST). It is unclear whether the difference between *AtFUL* and *CrFUL* action reflects differences in these genes or the species context. For example, *AtFUL* and *CrFUL* might both act as general valve growth and identity factors and it is other factors, which differ between the species, that are responsible for controlling the growth pattern that leads to the different fruit shapes. Candidates for these factors could be identified through screening for additional fruit shape mutants in *Capsella*.

The Brassicaceae family contains a richness of species with divergent fruit shapes. Some species have almost spherical fruits, such as *Neslia paniculata* (Francis and Warwick, 2003), a close relative of *Capsella*. Others have rounded fruits that are flattened laterally, such as *Lepidium campestre*, or in a medial orientation, such as *Alyssum maritimum* (Bowman, 2006) (Fig. 9). Using the models generated in this study, we found that the switch between lateral and medial flatness can be controlled simply by promoting K_par_ in the midvalve or replum regions, respectively (Fig. 9). This simple difference in growth patterns between the two types of fruit might explain the multiple incidents of evolutionary switches between medially and laterally flattened fruits in the Brassicaceae (Mummenhoff et al., 2005). As in *Arabidopsis* and *Capsella*, timing of regional anisotropic growth patterns might also be an important parameter in the coordination of fruit growth in other Brassicaceae members. In *A. linifolium* the gynoecium has a rounded cross-section before fertilisation and then becomes flattened medially during post-fertilisation growth. This might be achieved through promotion of K_par_ in the replum during the late phase.

It is possible that constraints, such as crossing connections between fruit walls, also contribute to fruit shape. A limitation of the modelling software used here is that such internal cross-connections are not modelled. However, even with this limitation, simple modulations of the model presented here can broadly account for the growth dynamics of *Capsella* and *Arabidopsis* and the variety of fruit shapes of other Brassicaceae species. Our model raises many further questions, including how regions such as the midvalve are defined and growth patterns established. Therefore, the model provides a simplified framework for fruit development that can be further tested experimentally to provide insight into the specific controls of growth phases and regional dynamics.

## MATERIALS AND METHODS

### Plant material and growth

*Arabidopsis thaliana* ecotype Col-0 and *Capsella rubella* Cr22.5 were used in all experiments. The *atful-2* mutant is in the Col-0 background. Plants were grown in glasshouse conditions at ∼22°C, 16 h photoperiod. Plants for clonal analysis and growth curve were grown in controlled environment rooms at 20°C (*A. thaliana*) or 22°C (*C. rubella*) in long-day conditions (8 h dark and 16 h light under fluorescent light at a photon fluence rate of 100 mmol m^−2^ s^−1^) and 80% humidity.

*Alyssum maritimum* fruits were a kind gift from J. M. Stacey and collected in her garden.

*Arabidopsis* and *Capsella* were transformed by floral dipping as described (Clough and Bent, 1998). Heat shocking of plant inflorescences was performed as described in the supplementary Materials and Methods.

### Growth curve

*Capsella* and *Arabidopsis* plants were grown on soil in a controlled environment room. The plants were standardised by selecting those at a similar stage. Whole inflorescences were collected and fixed for propidium iodide staining at 2-day intervals at about the same time of day, starting from 19-32 days after sowing. This method was developed from a protocol for staging *Arabidopsis* flower buds (Sauret-Gueto et al., 2013). Further details are provided in the supplementary Materials and Methods.

### Clonal analysis

Transgenic *C. rubella* Cr22.5 lines expressing pBOB (Wachsman et al., 2011) or HS-Cre (Gallois et al., 2002) were prepared. Lines with single copies of CrHS-Cre and CrBOB were crossed. For details, see the supplementary Materials and Methods.

### Computational modelling

For details of each of the models see the supporting model description and Table S4 in the supplementary Materials and Methods. All modelling was carried out with GPT-framework, implemented in the MATLAB (MathWorks) toolbox GFtbox (Kennaway et al., 2011) (http://cmpdartsvr3.cmp.uea.ac.uk/wiki/BanghamLab/index.php/GFtbox).

### Optical projection tomography

Samples were collected in 100% ethanol and rehydrated (80%, 60%, 40%, 20% ethanol, twice in H_2_O for 30 min each at room temperature) before being embedded in 1% low melting point agarose as described (Sharpe et al., 2002). Mounted specimens were dehydrated overnight in 100% methanol and cleared for 24 h in a 1:2 mixture of benzyl alcohol and benzyl benzoate (Sigma-Aldrich). Specimens smaller than 1 cm in width were scanned with a prototype OPT device as described previously (Lee et al., 2006). Specimens 1-2 cm in width were scanned using a Bioptonics 3001 scanner.

To visualise the OPT scans in 3D the freely available software package VolViewer (http://cmpdartsvr3.cmp.uea.ac.uk/wiki/BanghamLab/index.php/Software#Viewing_and_measuring_volume_images:_VolViewer) was used.

### Scanning electron microscopy

Samples were fixed in FAA (50% ethanol, 5% acetic acid, 3.7% formaldehyde) for 24 h, washed in 50% ethanol, dehydrated in an ethanol series (50%, 70%, 80%, 90%, four times in 100% ethanol for 30 min each at room temperature), and critical point dried using a Leica EM CPD300. Gynoecia were dissected from dried samples and mounted on stubs for coating in gold using an Agar Scientific high-resolution sputter coater and imaged using a Zeiss Supra 55VP FEG scanning electron microscope.

## References

[DEV135327C1] Alonso-Cantabrana H., Ripoll J. J., Ochando I., Vera A., Ferrandiz C. and Martinez-Laborda A. (2007). Common regulatory networks in leaf and fruit patterning revealed by mutations in the Arabidopsis ASYMMETRIC LEAVES1 gene. *Development* 134, 2663-2671. 10.1242/dev.02864 17592013

[DEV135327C2] Alvarez J. P., Goldshmidt A., Efroni I., Bowman J. L. and Eshed Y. (2009). The NGATHA distal organ development genes are essential for style specification in Arabidopsis. *Plant Cell* 21, 1373-1393. 10.1105/tpc.109.065482 19435933 PMC2700527

[DEV135327C3] Bowman J. L. (2006). Molecules and morphology: comparative developmental genetics of the Brassicaceae. *Plant System. Evol.* 259, 199-215. 10.1007/s00606-006-0419-8

[DEV135327C4] Clough S. J. and Bent A. F. (1998). Floral dip: a simplified method for *Agrobacterium*-mediated transformation of *Arabidopsis thaliana*. *Plant J.* 16, 735-743. 10.1046/j.1365-313x.1998.00343.x 10069079

[DEV135327C5] Dale E. E. (1925). Inheritance of fruit length in Capsicum. *Michigan Acad. Sci. Arts Lett.* 9, 89-110.

[DEV135327C6] Dinneny J. R., Weigel D. and Yanofsky M. F. (2005). A genetic framework for fruit patterning in *Arabidopsis thaliana*. *Development* 132, 4687-4796. 10.1242/dev.02062 16192305

[DEV135327C7] Emerson R. A. and East E. M. (1913). The inheritance of quantitative characters in maize. *Nebraska Agric. Expt. Sta. Res. Bull.* 2, 1-120.

[DEV135327C8] Ferrándiz C., Pelaz S. and Yanofsky M. F. (1999). Control of carpel and fruit development in Arabidopsis. *Annu. Rev. Biochem.* 68, 321-354. 10.1146/annurev.biochem.68.1.321 10872453

[DEV135327C9] Ferrándiz C., Liljegren S. J. and Yanofsky M. F. (2000). Negative regulation of the SHATTERPROOF genes by FRUITFULL during Arabidopsis fruit development. *Science* 289, 436-438. 10.1126/science.289.5478.436 10903201

[DEV135327C10] Francis A. and Warwick S. I. (2003). The biology of Canadian weeds. 120. Neslia paniculata (L.) Desv. *Can. J. Plant Sci.* 83, 441-451. 10.4141/P02-076

[DEV135327C11] Freeman G. F. (1919). The heredity of quantitative characters in wheat. *Genetics* 4, 1-93. 17245919 10.1093/genetics/4.1.1 PMC1200453

[DEV135327C12] Gallois J. L., Woodward C., Reddy G. V. and Sablowski R. (2002). Combined SHOOT MERISTEMLESS and WUSCHEL trigger ectopic organogenesis in Arabidopsis. *Development* 129, 3207-3217. 12070095 10.1242/dev.129.13.3207

[DEV135327C13] Green A. A., Kennaway J. R., Hanna A. I., Bangham J. A. and Coen E. (2010). Genetic control of organ shape and tissue polarity. *PLoS Biol.* 8, e1000537 10.1371/journal.pbio.1000537 21085690 PMC2976718

[DEV135327C14] Gu Q., Ferrandiz C., Yanofsky M. F. and Martienssen R. (1998). The *FRUITFULL* MADS-box gene mediates cell differentiation during Arabidopsis fruit development. *Development* 125, 1509-1517. 9502732 10.1242/dev.125.8.1509

[DEV135327C15] Hay A. and Tsiantis M. (2006). The genetic basis for differences in leaf form between *Arabidopsis thaliana* and its wild relative *Cardamine hirsuta*. *Nat. Genet.* 38, 942-947. 10.1038/ng1835 16823378

[DEV135327C16] Hilbert D. and Cohn-Vossen S. (1999). *Geometry and the Imagination*. New York, USA: AMS Chelsea Publishing.

[DEV135327C17] Kennaway R., Coen E., Green A. and Bangham A. (2011). Generation of diverse biological forms through combinatorial interactions between tissue polarity and growth. *PLoS Comput. Biol.* 7, e1002071 10.1371/journal.pcbi.1002071 21698124 PMC3116900

[DEV135327C18] Kuchen E. E., Fox S., de Reuille P. B., Kennaway R., Bensmihen S., Avondo J., Calder G. M., Southam P., Robinson S., Bangham A. et al. (2012). Generation of leaf shape through early patterns of growth and tissue polarity. *Science* 335, 1092-1096. 10.1126/science.1214678 22383846

[DEV135327C19] Kuusk S., Sohlberg J. J., Eklund D. M. and Sundberg E. (2006). Functionally redundant SHI family genes regulate Arabidopsis gynoecium development in a dose-dependent manner. *Plant J.* 47, 99-111. 10.1111/j.1365-313X.2006.02774.x 16740146

[DEV135327C20] Langowski L., Stacey N. and Østergaard L. (2016). Diversification of fruit shape in the Brassicaceae family. *Plant Reprod.* 29, 149-163. 10.1007/s00497-016-0278-6 27016361

[DEV135327C21] Lee K., Avondo J., Morrison H., Blot L., Stark M., Sharpe J., Bangham A. and Coen E. (2006). Visualizing plant development and gene expression in three dimensions using optical projection tomography. *Plant Cell* 18, 2145-2156. 10.1105/tpc.106.043042 16905654 PMC1560903

[DEV135327C22] Liljegren S. J., Roeder A. H. K., Kempin S. A., Gremski K., Østergaard L., Guimil S., Reyes D. K. and Yanofsky M. F. (2004). Control of fruit patterning in Arabidopsis by INDEHISCENT. *Cell* 116, 843-853. 10.1016/S0092-8674(04)00217-X 15035986

[DEV135327C23] Monforte A. J., Diaz A., Cano-Delgado A. and van der Knaap E. (2014). The genetic basis of fruit morphology in horticultural crops: lessons from tomato and melon. *J. Exp. Botany* 65, 4625-4637. 10.1093/jxb/eru017 24520021

[DEV135327C24] Mummenhoff K., Al-Shehbaz I. A., Bakker F. T., Linder H. P. and Mühlhausen A. (2005). Phylogeny, morphological evolution, and speciation of endemic brassicaceae genera in the Cape flora of Southern Africa. *Ann. Missouri Botanical Garden* 92, 400-424.

[DEV135327C25] Pabon-Mora N., Ambrose B. A. and Litt A. (2012). Poppy APETALA1/FRUITFULL orthologs control flowering time, branching, perianth identity, and fruit development. *Plant Physiol.* 158, 1685-1704. 10.1104/pp.111.192104 22286183 PMC3320178

[DEV135327C26] Paran I. and van der Knaap E. (2007). Genetic and molecular regulation of fruit and plant domestication traits in tomato and pepper. *J. Exp. Botany* 58, 3841-3852. 10.1093/jxb/erm257 18037678

[DEV135327C27] Roeder A. H. K. and Yanofsky M. F. (2006). Fruit development in Arabidopsis. *The Arabidopsis Book* 4, e0075 10.1199/tab.0075 22303227 PMC3243326

[DEV135327C28] Roeder A. H. K., Ferrandiz C. and Yanofsky M. F. (2003). The role of the REPLUMLESS homeodomain protein in patterning the Arabidopsis fruit. *Curr. Biol.* 13, 1630-1635. 10.1016/j.cub.2003.08.027 13678595

[DEV135327C29] Romera-Branchat M., Ripoll J. J., Yanofsky M. F. and Pelaz S. (2012). The WOX13 homeobox gene promotes replum formation in the Arabidopsis thaliana fruit. *Plant J.* 73, 37-49. 10.1111/tpj.12010 22946675

[DEV135327C30] Sauret-Gueto S., Schiessl K., Bangham A., Sablowski R. and Coen E. (2013). JAGGED controls arabidopsis petal growth and shape by interacting with a divergent polarity field. *PLoS Biol.* 11, e1001550 10.1371/journal.pbio.1001550 23653565 PMC3641185

[DEV135327C31] Scutt C. P., Vinauger-Douard M., Fourquin C., Finet C. and Dumas C. (2006). An evolutionary perspective on the regulation of carpel development. *J. Exp. Bot.* 57, 2143-2152. 10.1093/jxb/erj188 16720607

[DEV135327C32] Seymour G. B., Østergaard L., Chapman N. H., Knapp S. and Martin C. (2013). Fruit development and ripening. *Annu. Rev. Plant Biol.* 64, 219-241. 10.1146/annurev-arplant-050312-120057 23394500

[DEV135327C33] Sharpe J., Ahlgren U., Perry P., Hill B., Ross A., Hecksher-Sorensen J., Baldock R. and Davidson D. (2002). Optical projection tomography as a tool for 3D microscopy and gene expression studies. *Science* 296, 541-545. 10.1126/science.1068206 11964482

[DEV135327C34] Shull G. H. (1914). Duplicate genes for capsule-form in *Capsella bursa-pastoris*. *Z. Abst. u. Vererbl.* 12, 97-149.

[DEV135327C35] Sinnott E. W. (1935). Evidence for the existence of genes controlling shape. *Genetics* 20, 12-21. 17246742 10.1093/genetics/20.1.12 PMC1208568

[DEV135327C36] Sinnott E. W. and Kaiser S. (1934). Two types of genetic control over the development of shape. *Bull. Torrey Bot. Club* 61, 1-7. 10.2307/2481029

[DEV135327C37] Slotte T., Hazzouri K. M., Agren J. A., Koenig D., Maumus F., Guo Y.-L., Steige K., Platts A. E., Escobar J. S., Newman L. K. et al. (2013). The *Capsella rubella* genome and the genomic consequences of rapid mating system evolution. *Nat. Genet.* 45, 831-835. 10.1038/ng.2669 23749190

[DEV135327C38] Sluis A. and Hake S. (2015). Organogenesis in plants: initiation and elaboration of leaves. *Trends Genet.* 31, 300-306. 10.1016/j.tig.2015.04.004 26003219

[DEV135327C39] Tanksley S. D. (2004). The genetic, developmental, and molecular bases of fruit size and shape variation in tomato. *Plant Cell* 16, S181-S189. 10.1105/tpc.018119 15131251 PMC2643388

[DEV135327C40] Trigueros M., Navarrete-Gomez M., Sato S., Christensen S. K., Pelaz S., Weigel D., Yanofsky M. F. and Ferrandiz C. (2009). The NGATHA genes direct style development in the Arabidopsis gynoecium. *Plant Cell* 21, 1394-1409. 10.1105/tpc.109.065508 19435937 PMC2700528

[DEV135327C41] von Goethe J. W. (1790). *Versuch die Metamorphose der Pflanzen zu Erklären*. Gotha, Germany: C. W. Ettinger.

[DEV135327C42] Wachsman G., Heidstra R. and Scheres B. (2011). Distinct cell-autonomous functions of *RETINOBLASTOMA-RELATED* in *Arabidopsis* stem cells revealed by the Brother of Brainbow clonal analysis system. *Plant Cell* 23, 2581-2591. 10.1105/tpc.111.086199 21742994 PMC3226226

[DEV135327C43] Wang S., Lu G., Hou Z., Luo Z., Wang T., Li H., Zhang J. and Ye Z. (2014). Members of the tomato FRUITFULL MADS-box family regulate style abscission and fruit ripening. *J. Exp. Bot.* 65, 3005-3014. 10.1093/jxb/eru137 24723399 PMC4071821

[DEV135327C44] Xing S., Salinas M., Garcia-Molina A., Hohmann S., Berndtgen R. and Huijser P. (2013). SPL8 and miR156-targeted SPL genes redundantly regulate Arabidopsis gynoecium differential patterning. *Plant J.* 75, 566-577. 10.1111/tpj.12221 23621152

